# Prognostic Biomarkers for Survival in Nasopharyngeal Carcinoma: A Systematic Review of the Literature

**DOI:** 10.3390/cancers14092122

**Published:** 2022-04-24

**Authors:** Kazi Anisha Islam, Larry Ka-Yue Chow, Ngar Woon Kam, Ying Wang, Chi Leung Chiang, Horace Cheuk-Wai Choi, Yun-Fei Xia, Anne Wing-Mui Lee, Wai Tong Ng, Wei Dai

**Affiliations:** 1 Department of Clinical Oncology, University of Hong Kong, Hong Kong, China; kaislam@hku.hk (K.A.I.); u3523052@connect.hku.hk (L.K.-Y.C.); yvonnekam@hksccb.hk (N.W.K.); chiangcl@hku.hk (C.L.C.); hcchoi@hku.hk (H.C.-W.C.); awmlee@hku.hk (A.W.-M.L.); 2 Laboratory for Synthetic Chemistry and Chemical Biology, Hong Kong, China; 3 Department of Radiation Oncology, Sun Yat-sen University Cancer Centre, Guangzhou 510060, China; wangying2@sysucc.org.cn (Y.W.); xiayf@sysucc.org.cn (Y.-F.X.); 4 Center of Clinical Oncology, University of Hong Kong-Shenzhen Hospital, Shenzhen 518009, China

**Keywords:** nasopharyngeal carcinoma, prognosis, biomarkers, survival, systematic review

## Abstract

**Simple Summary:**

Nasopharyngeal carcinoma (NPC) is an important Epstein–Barr virus (EBV)-associated malignancy. Currently, tumor–nodes–metastases (TNM) staging and tumor markers, such as EBV DNA, are widely used for prognostication; however, the identification of novel molecular biomarkers, especially related to genetics and epigenetics alterations of patients, can potentially provide additional prognostic repertoire to the existing clinical parameters and biomarkers; hence, provide a robust method for risk stratification and effective personalized therapy. This systematic review identifies seventeen such important molecular biomarkers, including germline single-nucleotide polymorphisms (SNP), methylation, microRNAs, mutation, and gene signatures in cancer and immune cells, with strong evidence and a low risk of bias, which can be used to predict NPC outcome. It also highlights the clinical need for a comprehensive evaluation of multiple types of biomarkers in a multicenter study. The biomarkers identified are involved in various signaling pathways, which could be exploited as therapeutic targets in a clinical setting.

**Abstract:**

This systematic review aims to identify prognostic molecular biomarkers which demonstrate strong evidence and a low risk of bias in predicting the survival of nasopharyngeal carcinoma (NPC) patients. The literature was searched for on PubMed to identify original clinical studies and meta-analyses which reported associations between molecular biomarkers and survival, including ≥150 patients with a survival analysis, and the results were validated in at least one independent cohort, while meta-analyses must include ≥1000 patients with a survival analysis. Seventeen studies fulfilled these criteria—two studies on single nucleotide polymorphisms (SNPs), three studies on methylation biomarkers, two studies on microRNA biomarkers, one study on mutational signature, six studies on gene expression panels, and three meta-analyses on gene expressions. The comparison between the hazard ratios of high-risk and low-risk patients along with a multivariate analysis are used to indicate that these biomarkers have significant independent prognostic values for survival. The biomarkers also indicate a response to certain treatments and whether they could be used as therapeutic targets. This review highlights that patients’ genetics, epigenetics, and signatures of cancer and immune cells in the tumor microenvironment (TME) play a vital role in determining their survival.

## 1. Introduction

Nasopharyngeal carcinoma (NPC) is a rare but important malignancy, accounting for 133,354 new cases and 80,008 deaths globally in 2020 [1]. It has a unique geographical distribution with a much higher incidence in Southern China, Southeast Asia, the Arctic, parts of North Africa, and the Middle East [2,3], whereas it is conspicuously uncommon in Caucasian populations. According to the fourth edition of the World Health Organization (WHO) classification, NPC is classified as a nonkeratinizing, keratinizing, and basaloid squamous cell carcinoma; the nonkeratinizing tumors are further sub-categorized as undifferentiated or differentiated [4]. Staging is dependent on the anatomical extent of infiltration by the primary tumor, the involvement of lymph nodes, and the existence of distant metastasis. In the endemic regions for NPC, the commonest subtype is the nonkeratinizing tumor, which is almost unanimously associated with Epstein-Barr virus (EBV) [4]. Besides a latent infection of EBV, other risk factors for the carcinogenesis of NPC include the consumption of preserved foods such as salted fish [5], cigarette smoking, and the consumption of alcohol [6]. The gender ratio for NPC is interestingly skewed: the incidence is higher in men than women (3:1) [7].

According to a large multicenter study in Hong Kong, the 5-year and 8-year overall survival rates during 2001 to 2010 were 78.2% and 68.5%, respectively [8]. Currently, the tumor–nodes–metastases (TNM) staging method [9], based entirely on the anatomical disease extent, is the gold standard for risk group stratification, and it is the key factor to guide management plans. However, due to the intrinsic tumor heterogeneity and various host factors, there exists a spectrum of variation in prognosis within each stage of the disease. Many studies on the significance of various clinical factors have been published. Our group reported a previous systematic review on conventional NPC prognostic factors [10], including the EBV-DNA level, primary gross tumor volume (GTV), nodal GTV, neutrophil–lymphocyte ratio (NLR), C-reactive protein (CRP)/albumin ratio, anemia, platelet count, lactate dehydrogenase (LDH), and maximum standardized uptake value (SUVmax) of the primary tumor and total lesion glycolysis (TLG). However, with the increasing understanding of molecular aberrations and genetic alterations, it would be valuable to assess if we can further introduce the use of molecular stratification, striving to incorporate molecular/genetic signature into the anatomical staging system and conventional clinical biomarkers to provide a more precise risk estimation and individualized treatment strategy [11].

The primary objective of this systematic review is to identify novel molecular prognostic biomarkers with strong evidence that they could be predictive of NPC survival and could be used in conjunction with the existing anatomical staging system and conventional biomarkers. These biomarkers are often implicit in various signaling pathways, which could also be exploited as potential therapeutic targets.

## 2. Materials and Methods

### 2.1. Study Protocol and Criteria

The Preferred Reporting Items for Systematic Reviews and Meta-Analysis (PRISMA) guideline [12] was followed to conduct the review. A systematic search of the literature reported between 1st January 2011 and 9th September 2021 was performed on PubMed. The search was conducted using the terms “staging” or “TNM” or “prognostic” and “biomarker” and “nasopharyngeal (carcinoma, cancer, or neoplasm)”. Both English and Chinese literature was accepted, while unpublished studies and studies in other languages were not included. The full search strategy is presented in Table S1 in Supplementary Materials. Studies were included if they met the following criteria: (1) original clinical studies (prospective/retrospective) on the association between prognostic factors and survival; (2) ≥150 NPC patients; (3) results validated in at least one independent cohort. In the reporting of meta-analyses, studies were included if they met the following criteria: (1) ≥1000 cases and (2) analyses on the association between prognostic factors and survival. The primary endpoint of the assessment was overall survival (OS), secondary endpoints, including disease-free survival (DFS), progression-free survival (PFS), or distant metastasis-free survival (DMFS) and locoregional recurrence-free survival (LRRFS) were also incorporated if they were reported by the original study. The data elements of eligible studies were analyzed and summarized.

### 2.2. Inclusion Process

The following studies were excluded from the initial search after screening their titles and citations: abstracts, reviews or systematic reviews, editorials, case reports, duplicated studies, letters, book chapters, basic science studies, retracted articles, and other nonrelevant studies. Studies included in our previous systematic review by Chiang et al. on conventional clinical prognostic factors [10], including EBV-DNA level, primary GTV, nodal GTV, NLR, CRP/albumin ratio, anemia, platelet count, LDH, and SUVmax of the primary tumor and TLG were also excluded to avoid duplication. The remaining studies, which included original clinical studies, either prospective or retrospective, were further examined to confirm adequate sample size and whether they reported survival outcomes with sufficient data (hazard ratios (HR), 95% confidence intervals, *p*-values).

Two independent reviewers (W.D. and K.A.I.) performed the first review to exclude ineligible studies by screening the titles and citations. They also determined whether the prognostic biomarkers were addressed by Chiang et al. [10]. A consensus was reached, and discussions were conducted regarding the relevance of the titles and citations in the case of any disagreements. A third independent reviewer (L.K.-Y.C.) was added to the team, and all three independent reviewers screened for studies that demonstrated an association with survival, contained relevant data with an adequate sample size in the discovery set, and reported at least one independent validation cohort.

The studies included were assessed for risk of bias using the Quality in Prognosis Studies (QUIPS) tool [13]. The tool helps to assess bias in six domains—study participation, prognostic factor measurement, outcome measurement, statistical analysis and reporting, study confounding, and study attrition. Each domain comprised 3–7 prompting items that guided the reviewers. If >80% of the prompting items were marked “yes” or “partial”, the risk of bias was low (score = 3). If 20–80% of the prompting items were marked “yes” or “partial”, the risk of bias was moderate (score = 2). If <20% of the prompting items were marked “yes” or “partial”, the risk of bias was high (score = 1). If the total score was >15, the risk of bias was low and, hence, the quality of the study was high. If the total score was 9–15, the risk of bias was moderate and, hence, the quality of the study was moderate. If the total score was <9, the risk of bias was high and, hence, the quality of the study was low. Only studies of high- and moderate-quality were retained.

The study protocol and inclusion process outlined above was registered on PROSPERO. Details of the protocol for this systematic review can be accessed at https://www.crd.york.ac.uk/prospero/display_record.php?ID=CRD42022302142 [14] (accessed on 1 March 2022). The PROSPERO database was also searched, and it was identified that a systematic review on the molecular/genetic prognostic biomarkers for survival in NPC did not exist, which served the purpose of avoiding duplication.

## 3. Results 

**Figure 1:**
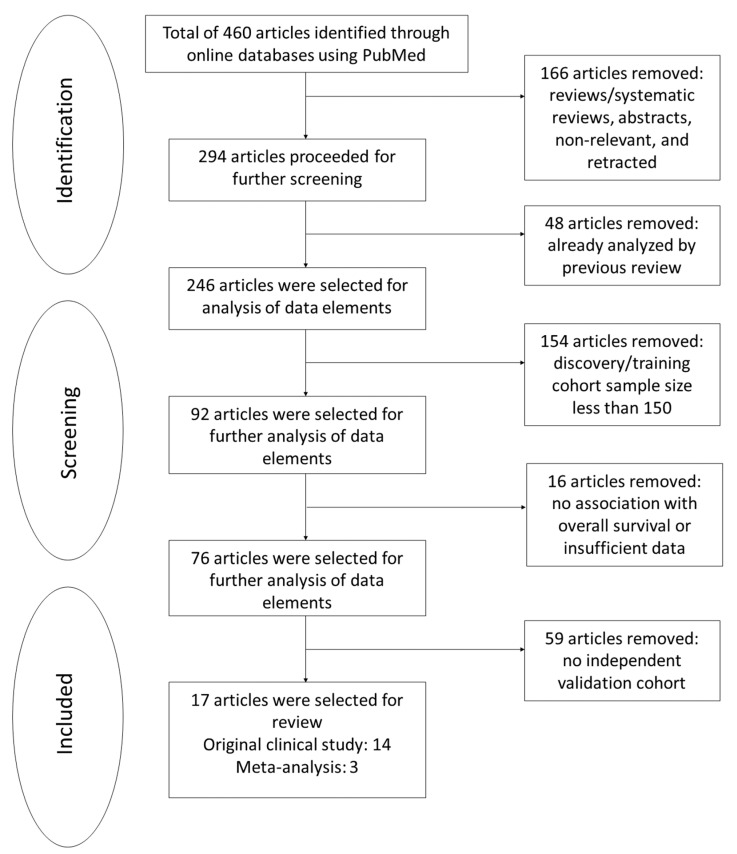
Inclusion process and criteria. The search on PubMed output 460 results, which were subject to a stepwise filtration process. Reviews/systematic reviews, abstracts, nonrelevant and retracted articles were excluded, followed by removing articles which were previously covered by another systematic review. The remaining eligible studies were filtered on whether they had an adequate sample size, whether survival was reported and whether the results were validated in an independent cohort. The process yielded 17 articles—14 original clinical studies and 3 meta-analyses that met the criteria outlined above.

Of 460 studies screened, 166 were reviews (26), systematic reviews (7), abstracts (3), or retracted and nonrelevant articles (130). Among them, 48 articles were excluded because they overlapped with our previous systematic review by Chiang et al. [10]. From the remaining 246 articles, 154 were further excluded due to an inadequate sample size in the discovery set, 16 for insufficient data or without survival analyses, and 59 for lacking a validation cohort. The full inclusion process is outlined in Figure 1. The risk scores, adjusted HR, 95% CI, *p*-values, and multivariate clinical parameters were summarized systematically in Table S2 in Supplementary Materials.

Seventeen studies comprising 189,401 patients with survival outcomes were eventually selected, all showing strong evidence and high quality using the risk of bias assessment: fourteen studies from original clinical studies and three studies from meta-analyses. Prognostic factors from these original clinical studies included two studies on single-nucleotide polymorphisms (SNPs), three studies on methylation biomarkers, two studies on microRNA biomarkers, one study on mutational signature, and six studies on gene expression panels. In addition, prognostic factors from the meta-analysis studies included three studies on gene expressions. 

### 3.1. Single-Nucleotide Polymorphisms (SNPs)

#### 3.1.1. rs3740194 in CUGBP Elav-like Family Member 2 Gene

The genotype AA for the rs3740194 SNP in the CUGBP Elav-Like Family Member 2 (*CELF2*) gene was significantly associated with both inferior OS (AA vs. AG + GG; HR = 1.53, 95% CI = 1.23–1.89; *p* = 1.30 × 10^4^) and DMFS (AA vs. AG + GG; HR = 1.60; 95% CI = 1.26–2.02; *p* = 8.87 × 10^5^) compared with the genotypes AG and GG after multivariate adjustment with age, gender, T stage, N stage, overall stage, and treatment with radiotherapy and different types of chemotherapy [15]. The multivariate analysis of the NPC patient samples confirmed that this association was independent of the clinical parameters. *CELF2* could potentially act as a tumor suppressor. It could also cause a mitotic catastrophe in cancer cells and prevent the translation of proteins involved in lymph node invasion and distant metastasis.

#### 3.1.2. rs1131636 in Replication Protein A1 Gene

The rs1131636 SNP at the replication protein A1 (*RPA1*) gene was significantly associated with OS (HR = 1.33; 95% CI = 1.20–1.47; *p* = 6.31 × 10^−8^) and DMFS (HR = 1.16; 95% CI = 1.05–1.28; *p* = 0.0033) in patients with NPC in the combined cohort [16]. Covariates in the multivariable analysis included age, gender, clinical stage, intensity modulated radiotherapy (IMRT), induction chemotherapy (ICT), concurrent chemoradiotherapy (CCRT), and adjuvant chemotherapy (ACT). The study showed that patients carrying the rs1131636-[CC] genotype had a 5-year OS rate of 90.3% (95% CI = 88.0–92.5%), whereas those who carried other genotypes had a shorter 5-year OS rate of 83.4% (95% CI = 82.0–84.9%). In further in vitro experiments in a human normal cell line and NPC cell lines, when separately transfected with the rs1131636-[C] and rs1131636-[T] constructs, the cells with the C variant exhibited lower levels of luciferase activity compared with the cells with the T variant. This might explain why patients carrying the CT/TT genotype could potentially accommodate the growth of an aggressive tumor subtype. miR-1253 was also observed to target the C allele of rs1131636, reduce luciferase activity and inhibit other protein translations. Furthermore, the overexpression of *RPA1* has been linked to enhanced cell proliferation, migration, invasion, and radiation resistance of NPC cells.

### 3.2. microRNAs (miRNAs)

#### 3.2.1. miR-142-3p, miR-29c, miR-26a, miR-30e, and miR-93

A risk score formula was derived based on expression values of five miRNAs: miR-142-3p, miR-29c, miR-26a, miR-30e, and miR-93 [17]. The expression levels of the protective biomarkers—miR-142-3p, miR-29c, miR-26a, and miR-30e—were positively associated, while the expression level of the risk biomarker miR-93 was inversely associated with DFS, DMFS, and OS. After multivariate adjustment for age, sex, TNM stage, WHO pathological type, radiotherapy period interruptions, radiotherapy boosting, concurrent chemotherapy (CCT), viral capsid antigen immunoglobin-A (VCA IgA), and early antigen immunoglobin-A (EA IgA), it was observed that the high-risk scores were associated with a shorter DFS (HR = 3.16; 95% CI = 1.65–6.04; *p* = 0.0011), DMFS (HR = 2.39; 95% CI = 1.05–5.42; *p* = 0.037), and OS (HR = 3.07; 95% CI = 1.34–7.01; *p* = 0.0082). The study also reported that none of the five-minus-one miRNA signatures, which only included four miRNAs, showed the significant associations with DFS, DMFS, and OS. The study also noted that the low-risk patients expressed higher levels of the protective miRNAs. Among the patients with advanced stages, those with high-risk scores had a poorer response to CCRT and shorter survival. Combining the miRNA signature with the TNM staging system provided a better prognosis than the TNM staging alone in all the study cohorts; thus, enabling clinicians to approach treatments more systemically to yield better outcomes.

#### 3.2.2. miR-22, miR-572, miR-638, and miR-1234

A risk score formula was derived based on the expression values of four miRNAs: miR-22, miR-572, miR-638, and miR-1234 [18]. The expression levels of the protective biomarkers (miR-22, miR-572, and miR-638) were positively associated with OS, and the expression level of the adverse biomarker (miR-1234) was inversely associated with OS. After a multivariate adjustment at the TNM stage, it was observed that patients with higher risk scores had a significantly shorter OS (HR = 2.40; 95% CI = 1.71–3.37; *p* < 0.001) and DMFS (HR = 3.31; 95% CI = 2.18–5.02; *p* < 0.001) in the training, validation, and combined cohorts compared with patients with lower risk scores. The survival prediction of this four-miRNA signature was independent of the TNM staging system and provided a higher prognostic value when combined with it. By stratifying patients into high-risk and low-risk groups according to their TNM stages and risk scores combined, a more precise decision could be determined regarding the intensity of treatment and thus, potentially improve the treatment outcomes. This serum miRNA signature may also be responsible for intercellular communications that influence how NPC might progress and develop. Further investigations are suggested to determine if this four-miRNA signature could act as a therapeutic target.

### 3.3. DNA Methylation

#### 3.3.1. Hypermethylated Gene Panel (WNT Inhibitory Factor 1, Ubiquitin C-Terminal Hydrolase L1, Ras Association Domain Family Member 1, Cyclin A1, Tumor Protein P73, and Secreted Frizzled Related Protein 1)

Samples were classified into high and low methylation groups according to average Z-scores measured from the frequencies of methylation of the six genes—WNT Inhibitory Factor 1 (*WIF1*), Ubiquitin C-Terminal Hydrolase L1 (*UCHL1*), Ras Association Domain Family Member 1 (*RASSF1A*), Cyclin A1 (*CCNA1*), Tumor Protein P73 (*TP73*), and Secreted Frizzled Related Protein 1 (*SFRP1*) at CpG sites [19]. High methylation was significantly associated with a shorter DFS (HR = 2.08; 95% CI = 1.17–3.68; *p* = 0.013) and OS (HR = 1.83; 95% CI = 1.01–3.31; *p* = 0.046) after multivariate adjustment for sex, age, TNM stage, WHO pathology type, and CCRT. The multivariate analysis of the samples in all cohorts revealed that the methylation gene panel was an independent prognostic factor of DFS and OS. Further studies on the effect of methylation within the TNM stages showed that among patients with advanced stages, those with high methylation had a shorter DFS (HR = 2.19; 95% CI = 1.52–3.16; *p* < 0.001) and OS (HR = 2.35; 95% CI = 1.58–3.49; *p* < 0.001). These associations could be attributed to the various pathways that the six genes are involved in to induce apoptosis and cell cycle arrest, inhibit cell growth, invasion, angiogenesis, and cell migration of cancer cells. Hence, the methylation status of the abovementioned genes could be a significant prognostic biomarker for tumor progression in NPC.

#### 3.3.2. Hypermethylation of TNF Alpha-Induced Protein 8-like 3

Methylation levels of the CpG site (cg05905176) on the TNF Alpha-Induced Protein 8-Like 3 (*TIPE3*) gene were significantly higher in the NPC cell lines compared with normal nasopharyngeal epithelial cells (NPEC) [20]. As a result, *TIPE3* expression was significantly downregulated in the NPC cells. Using the receiver operating characteristic (ROC) curve analysis, patient samples were categorized into high and low methylation levels at this selected CpG site on *TIPE3*. Higher *TIPE3* methylation levels were associated with a shorter DFS (HR = 1.71; 95% CI = 1.01–2.89; *p* = 0.045), DMFS (HR = 2.69; 95% CI = 1.21–5.96; *p* = 0.015), and OS (HR = 1.80; 95% CI = 1.02–3.16; *p* = 0.041). It was also noted that the prognostic value of the *TIPE3* methylation was independent of other clinical characteristics, including age, sex, WHO type, VCA IgA, EA IgA, and TNM stage. The suppression of *TIPE3* expression enhanced NPC cell proliferation, migration, and invasion. *TIPE3* hypermethylation in the CpG island (CGI) has frequently been observed and contributes to the silencing of *TIPE3* expression in many cancer types, including NPC. Since CGI methylation is partially reversible, it can be a potential prognostic biomarker and therapeutic target to treat NPC.

#### 3.3.3. Hypermethylation of HOP Homeobox

Patients with higher (>13.5%) HOP Homeobox (*HOPX*) methylation levels were significantly associated with a shorter OS (HR = 2.13; 95% CI = 1.15–3.93; *p* = 0.016), DFS (HR = 1.96; 95% CI = 1.12–3.43; *p* = 0.019), and DMFS (HR = 2.75; 95% CI = 1.26–6.03; *p* = 0.011) in the study cohorts including a total of 443 patients [21]. The multivariate analysis revealed that *HOPX* methylation was an independent prognostic factor after adjustment for age, sex, WHO type, VCA IgA, or EA IgA. Both in vitro and in vivo studies suggest that *HOPX* suppresses the epithelial-to-mesenchymal transition (EMT), invasion, and distant metastasis, and is relevant to cisplatin sensitivity. Therefore, the methylation and suppression effect of *HOPX* could be a potential therapeutic target and act as an important prognostic biomarker of NPC progression.

### 3.4. Mutational Signatures

#### COSMIC Mutational Signatures (Homologous Recombination Repair and Mismatch Repair)

The COSMIC signature 3, a type of BRCAness signature, and mismatch repair (MMR) signature were found to be independent prognostic factors in NPC [22]. In total, 22.7% of the NPC patients were found to contain both signatures. The multivariate analysis (in the age and TNM stage) showed that compared with other signature groups, cases with both signatures had significantly higher risks of death (HR = 8.9; 95% CI = 2.1–38; *p* = 0.003) in the validation cohort. BRCA2 DNA Repair-Associated (*BRCA2*) germline rare variants were observed to be independently associated with a shorter OS (HR = 1.9; 95% CI = 1.0–3.7; *p* = 0.046) and PFS (HR = 1.9; 95% CI = 1.0–3.4; *p* = 0.042). A further analysis demonstrated that a higher level of signature 3 was correlated to a high chromosome instability and a unique pattern in the methylation profile of the sample genomes. The EBV methylation profile of signature-positive cases exhibited distinct hypomethylated regions as well. Mutational signatures such as this could potentially contribute to aggressive phenotypes of NPC, making them more resistant to conventional treatment. The homologous recombination repair (HR) and MMR pathways resulting in these signatures might act as therapeutic targets in a subset of NPC patients.

### 3.5. Gene Signatures

#### 3.5.1. Eight-Signature Classifier (Patient Sex, EBV-Latent Membrane Protein 1, CD147, Caveolin-1, Phospho-p70 S6 Kinase, Matrix Metallopeptidase 11, Survivin, Secreted Protein Acidic, and Cysteine Rich)

A classifier was developed to combine gender with the expression of seven genes—EBV-latent membrane protein 1 (EBV-LMP1), CD147 (also known as BSG, an OK blood group antigen), caveolin-1 (also known as CAV1, a scaffolding protein within caveolar membranes), Phospho-p70 S6 Kinase (p-P70S6K), Matrix Metallopeptidase 11 (MMP11),survivin (also known as BIRC5, an apoptotic inhibitor), and Secreted Protein Acidic and Cysteine-Rich (SPARC; a cysteine-rich acidic matrix-associated protein) as prognostic biomarkers for NPC [23]. Patients were then classified as high-risk or low-risk. There was a significant difference in 5-year DSS rates between the high-risk and low-risk patients (87.0% vs. 37.7%, *p* < 0.001). After a multivariate adjustment, the classifier was independent of the clinical stage, age, and WHO histologic subtype, and the signature still demonstrated a five-fold difference in DSS between low-risk and high-risk groups (HR = 4.9; 95% CI = 3.0–7.9; *p* < 0.001).

#### 3.5.2. Immune Signature (PD-L1, CD163 Molecule, C-X-C Motif Chemokine Receptor 5, and CD117)

This immune signature revealed the intratumoural expression status of PD-L1, CD163, C-X-C Motif Chemokine Receptor 5 (CXCR5), and CD117, and classified patients into the high-risk and low-risk groups [24]. A regression model was established, which selected the optimal cut-off value. Compared to the low-risk groups, the patients in the high-risk group exhibited a shorter DMFS (HR = 4.297; 95% CI = 2.182–8.461; *p* < 0.0001) after the multivariable adjustment (including gender, age, TNM stage, Eastern Cooperative Oncology Group (ECOG) score, LDH, CRP, hemoglobin (Hg), and body mass index (BMI)). Moreover, in patients with a low immune signature score, induction chemotherapy followed by CCRT was associated with a longer DMFS (HR = 0.355; 95% CI = 0.147–0.857; *p* = 0.021) and PFS (HR = 0.590; 95% CI = 0.351–0.992; *p* = 0.047) compared to those treated with CCRT alone. It was also found that a nomogram incorporating the immune signature, N category, and Hg could accurately predict the risk of distant metastasis. However, the incorporation of the plasma EBV DNA level failed to improve the accuracy, possibly due to the correlation between the EBV and immune expression profile. This study provided useful information on PD-L1+ macrophages as biomarkers in predicting a response to checkpoint inhibitors. Expression levels of CXCR5 and CD117 could also be considered as intratumor targets in the future.

#### 3.5.3. Delta-like Canonical Notch Ligand 4 and Vascular Endothelial Growth Factor A Expression

There was a significant difference in 5-year DSS rates between patients with low and high expression levels of Delta-Like Canonical Notch Ligand 4 (DLL4) (65.1% vs. 34.8%, *p* = 0.004) [25]. The multivariable analysis (with age, gender, and TNM stage) showed that the DLL4 expression level was a significant independent prognostic factor for a shorter DSS (HR = 1.809; 95% CI = 1.380–2.370; *p* < 0.001). Moreover, distant metastatic lesions exhibited higher DLL4 protein levels than matched nasopharyngeal tissues. It was also found that there was a positive correlation between DLL4 and Vascular Endothelial Growth Factor A (VEGF) expression levels in NPC (r_s_ = 0.404, *p* < 0.001). Patients with a dual overexpression of DLL4 and VEGF had a significantly lower 5-year DSS rate compared to patients with a dual low expression (71.2% vs. 25.4%; *p* = 0.003). Since DLL4 expression could potentially be blocked through the DLL4-Notch pathway, it could act as a therapeutic target to suppress tumor growth and angiogenesis in NPC.

#### 3.5.4. Tumor-Infiltrating Lymphocytes

NPC has a rich infiltrating lymphocytes tumor microenvironment [26]. Wang et al. carried out a study to score all the mononuclear cells, including lymphocytes and plasma cells, and evaluated the intratumoral lymphocyte-infiltrating intensity (I-TLII) and stromal lymphocyte-infiltrating intensity (S-TLII) in NPC tumor tissues. Tumor-infiltrating lymphocytes (TILs) were evaluated by combining I-TLII and S-TLII [27]. The study found that high TILs (I-TLII > 10% and/or S-TLII > 70%) in patients were associated with a more favorable 5-year DFS compared to those with low TILs (87.0% vs. 72.4%; HR = 0.52; 95% CI = 0.36–0.75; *p* < 0.001). Similarly, the OS was significantly different between the two groups stratified by TIL quantities. The multivariable analysis (with TNM stage, radiotherapy technique, EBV DNA level, and LDH) demonstrated that the TIL quantity was an independent prognostic factor for DFS.

#### 3.5.5. 13 Gene-Signature (*YBX3*, *CBR3*, *CXCL10*, *CLASP1*, *DCTN1*, *FNDC3B*, *WSB2*, *LRIG1*, *GRM4*, *ANXA1*, *WNK1*, *HDLBP*, *POLR2M*)

Based on four cohorts of a total of 937 samples, a signature consisting of 13 differentially expressed genes, including the Y-Box Binding Protein 3 (*YBX3*), Carbonyl reductase 3 (*CBR3*), C-X-C Motif Chemokine Ligand 10 (*CXCL10*), Cytoplasmic Linker-Associated Protein 1 (*CLASP1*), Dynactin Subunit 1 (*DCTN1*), Fibronectin Type III Domain Containing 3B (*FNDC3B*), WD Repeat and SOCS Box Containing 2 (*WSB2*), Leucine-Rich Repeats and Immunoglobulin-Like Domains 1 (*LRIG1*), Glutamate Metabotropic Receptor 4 (*GRM4*), Annexin A1 (*ANXA1*), WNK Lysine-Deficient Protein Kinase 1 (*WNK1*), High-Density Lipoprotein Binding Protein (*HDLBP*), and RNA Polymerase II Subunit M (*POLR2M*), was found to be associated with DMFS in the patients with locoregionally advanced NPC [28]. Significant correlations were also observed between the distant metastasis gene signature (DMGN) and DFS and OS. Optimal cutoffs were determined, and patients were stratified as high or low risk based on these 13 gene expressions. In the validation cohort, the 5-year DMFS was significantly shorter in the high-risk group than in the low-risk group (HR = 2.98; 95% CI = 1.60–5.55; *p* = 0.00032). The DMGN remained a strong independent prognostic factor for DMFS after a multivariable adjustment by the clinicopathological variables. In the low-risk group, the patients received a CCRT-demonstrated longer DMFS (HR = 0.40; 95% CI = 0.19–0.83; *p* = 0.011), DFS (HR = 0.56; 95% CI = 0.34–0.9; *p* = 0.025), and OS (HR = 0.58; 95% CI = 0.35–0.97; *p* = 0.036) than those without CCRT. On the other hand, in the high-risk group, those with CCRT did not achieve significant benefit in DMFS and OS, suggesting that CCRT was an effective treatment for low-risk patients, but high-risk patients would require more aggressive forms of treatment to prevent distant metastasis. A nomogram (incorporating DMGN, sex, N category, plasma EBV DNA level, LDH, and CRP) was also constructed to predict distant metastasis in patients with locoregionally advanced NPC.

#### 3.5.6. Protein Tyrosine Phosphatase 4A2

The overexpression of protein tyrosine phosphatase 4A2 (*PTP4A2*) was significantly correlated with a poor OS (HR = 5.957; 95% CI: 4.157–8.853; *p* < 0.001) and DFS (HR = 4.349; 95% CI = 3.158–5.988; *p* < 0.001) [29]. The multivariable analysis (including age, gender, TNM stage, WHO type, VCA IgA, and EA IgA) revealed that the overexpression of *PTP4A2* was an independent adverse prognostic factor for OS (HR = 2.784; 95% CI = 3.490–7.351; *p* < 0.001) and DFS (HR = 3.669; 95% CI = 2.635–5.110; *p* < 0.001). In addition, the immunohistochemistry (IHC) analysis also demonstrated that PTP4A2 was overexpressed in 40.6% and 50.7% of NPC tissues in the training and validation cohorts, respectively. It was postulated that the *PTP4A2* gene is an oncogene which could be responsible for enhancing tumourigenesis and progression in NPC, similar to other types of human cancer such as breast cancer, colon cancer, and lung cancer. However, more functional studies need to be conducted to elucidate the signaling pathways affected by the overexpression of *PTP4A2* in NPC.

### 3.6. Biomarkers Identified from Meta-Analyses

The expression of Hypoxia Inducible Factor 1 Subunit Alpha (HIF-1α; a heterodimeric transcription factor in response to hypoxia) was significantly associated with a poor OS in retrospective studies (HR = 2.19; 95% CI = 1.53–3.10; *p* < 0.001) and PFS (HR = 1.72; 95% CI = 1.22–2.44; *p* = 0.002) in both prospective and retrospective studies [30]. The heterogeneity test demonstrated no significance (I^2^ = 0.0%; *p* = 0.566). Patients with high VEGF (a potent angiogenic factor) expression were also significantly associated with an inferior OS (HR = 2.07; 95% CI = 1.32–3.25) and DFS (HR = 5.99; 95% CI = 2.66–13.48) [31]. The positive expression of ERCC Excision Repair 1 (*ERCC1*; a DNA excision repair protein) also predicted a poor OS (HR = 1.77; 95% CI = 1.48–2.12; *p* < 0.001) and DFS (HR = 1.60; 95% CI = 1.43–1.79; *p* < 0.001) in NPC patients [32].

## 4. Discussion

**Figure 2:**
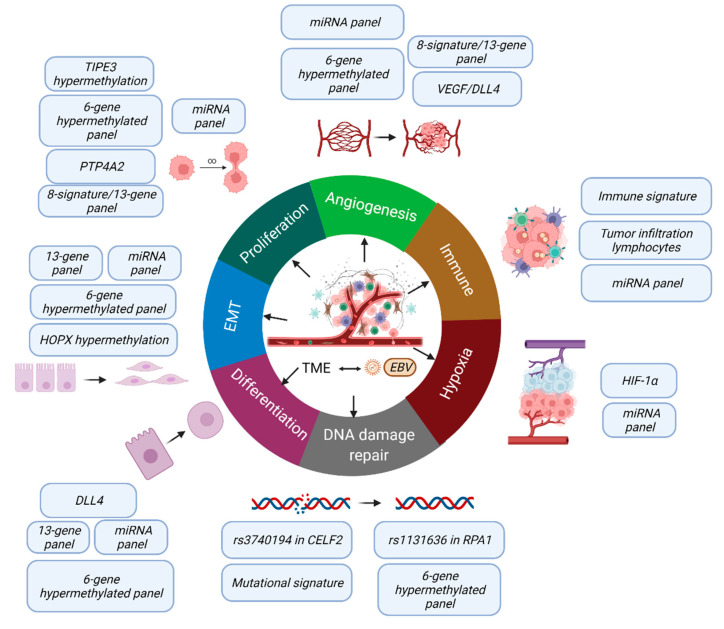
A summary of the biological functions involved in the biomarkers identified in this review. We identified the molecular changes for patients’ genetics (germline single-nucleotide polymorphisms (SNPs) and mutational signatures), epigenetics (DNA methylation and microRNA), and gene expression from cancer cells and immune cells in the tumor microenvironment (TME) in determining the clinical outcome for NPC. These molecular changes trigger various biological pathways to influence cancer proliferation, DNA damage repair, angiogenesis, hypoxia, epithelial-to-mesenchymal transition (EMT), differentiation, and immune response. Created with https://biorender.com/ [33] (accessed on 15 March 2022).

To the best of our knowledge, this systematic review of the literature is the most comprehensive review of novel molecular biomarkers with strong evidence and a low risk of bias for predicting NPC survival outcomes. The SNPs identified in this systematic review were the rs1131636 on the *RPA1* gene [16] and rs3740194 on the *CELF2* gene [15]. Methylation studies included the hypermethylated gene panel of six genes—*WIF1*, *UCHL1*, *RASSF1A*, *CCNA1*, *TP73*, and *SFRP1* [19], and the hypermethylation of the *TIPE3* [20] and HOPX [21] genes. The miRNA panels reported were the five-miRNA signature consisting of miR-142–3p, miR-29c, miR-26a, miR-30e, and miR-93 [17] and the four-miRNA signature consisting of miR-22, miR-572, miR-638, and miR-1234 [18]. The combined mutational signature 3 and MMR signature were included in this systematic review too [22]. Lastly, the gene expression signatures included were the 13-gene signature panel—*YBX3*, *CBR3*, *CXCL10*, *CLASP1*, *DCTN1*, *FNDC3B*, *WSB2*, *LRIG1*, *GRM4*, *ANXA1*, *WNK1*, *HDLBP*, and *POLR2M* [28]; the 8-signature classifier—sex, caveolin-1, CD147, EBV-LMP1, MMP11, p-P70S6K, survivin, and SPARC [23]; the immune signature panel—PD-L1, CD163, CXCR5, and CD117 [24], DLL4 with VEGF expression [25], tumor infiltrating lymphocytes (TILs) [27], and PTP4A2 [29]. Other prognostic biomarkers including the expression of the ERCC1 [32], VEGF [31] and HIF-1α [30] genes were also noted. This highlighted the critical and complex roles of patients’ genetics (germline SNPs and mutational signatures), epigenetics (DNA methylation and miRNA), and gene signatures from cancer cells and immune cells in thess tumor microenvironment in the clinical course of the disease. These molecular changes trigger various biological pathways to influence cancer proliferation, DNA damage repair, angiogenesis, hypoxia, EMT, differentiation, and the immune response (Figure 2), which directly affect patients’ prognosis.

Furthermore, a comprehensive evaluation of the prognostic value of multiple types of biomarkers identified in this review is prudent in the realm of precision medicine. In addition to the conventional multivariable survival analysis, the cutting-edge artificial intelligence technology could facilitate us the evaluation of these complex bioinformatics analyses and help identify the patterns for patients with unfavorable clinical outcomes. Recently, we utilized a deep learning method to establish a model for survival using the transcriptomic features and clinical parameters in hepatocellular carcinoma [34]. A multicenter study is currently ongoing to analyze the use of deep learning methods for developing a more precise prognostic system based on anatomical TNM and nonanatomical conventional clinical markers. The next level of refinement would be the further incorporation of the above identified molecular/genetic factors. Additionally, it is possible to establish the machine learning classifiers, such as random forest (RF), support vector machine, gradient boosting machine, conditional RF, neural network, naïve bayes, elastic net, and logistic regression, for risk stratification.

While this was an extensive review of novel biomarkers, several factors need to be considered. Firstly, due to the anatomical location of the nasopharynx, an adequate amount of tumor samples is often difficult to obtain; hence, clinical studies are often limited by small sample sizes. As a result, out of 246 eligible studies, only 92 studies were eligible in terms of a sample size of ≥150 as specified in this systematic review. This may undermine the extent of other relevant prognostic biomarkers. Secondly, many studies had no independent validation, and the robustness of the biomarkers could not be ascertained. This led to the removal of another 59 studies reported in the literature. Thirdly, all the studies analyzed in this review were carried out using tissue biopsies, which is a relatively more invasive approach. Recently, the use of noninvasive liquid biopsies, such as whole blood, plasma, or serum for the detection of circulating tumor DNA (ctDNA), cell-free DNA (cfDNA), and circulating tumor cells (CTCs), have been widely adopted for disease detection, monitoring, and prognosis of not only NPC, but also of other cancer types [35,36,37,38,39]. In fact, the circulating plasma EBV DNA measurement has been widely employed for the screening, early detection, risk stratification, and disease monitoring of NPC [40]. However, no standardized cut-off level has been identified due to the heterogeneity in the testing method [40]. It is also worth noting that the EBV DNA level reflects tumor load, but it does not provide any valuable information regarding tumor responsiveness to treatment and the emergence of resistant tumor clones. On the other hand, the molecular biomarkers described in this review not only provided useful complementary prognostic information to the TNM staging system but may also provide important insights into NPC pathogenesis and the identification of novel therapeutic targets.

Last, but not least, there is a need for future multicenter studies to rectify the prognostic value of these molecular biomarkers to ensure a robust clinical application. Fundamental scientific research is also needed to examine the mechanistic actions of these unfavorable prognostic biomarkers involving specific SNPs, the hypermethylation status, and the various mutational, miRNA, and gene expression signatures. In addition, caution must be exercised when applying the biomarkers identified by Guo et al. [15], Liu et al. [17], Liu et al. [18], Jiang et al. [19], Wang et al. [23], and Zhang et al. [25] for further evaluation, as these studies were carried out for more than five years on the publication date of this review without clinical application.

The pressing question is can we group together all these findings to develop a pragmatic panel with the greatest discriminating power. As the factors are involved in different pathways with their respective impact on tumor natural behavior (Figure 2), it is important to focus on the driver factor. For example, among the various factors that affect angiogenesis, which should be selected as the key prognostic factors and targets for therapy? This new direction for future study has been urgently awaited for in regard to advancing the goal of precision oncology.

## 5. Conclusions

This review identified multiple novel molecular biomarkers for predicting NPC prognosis and survival. These biomarkers were highly relevant in the disease pathways, including cancer proliferation, DNA damage repair, angiogenesis, hypoxia, EMT, differentiation, and the immune response. These complex processes and interactions among cancer cells, EBV, and tumor microenvironment were shown to be crucial in predicting outcomes. A multi-center study to comprehensively evaluate the prognostic value of these biomarkers together with other known prognostic factors utilizing AI technologies is recommended. In the future, with advanced genomic, epigenomic, and transcriptomic profiling technologies, a precision treatment strategy can be envisaged based on individualized molecular profiles. The biological pathways involved with these biomarkers can also be examined in functional studies as therapeutic targets for NPC treatment.

## Supplementary Materials

The following are available online at https://www.mdpi.com/article/10.3390/cancers14092122/s1, Table S1: search parameters on PubMed; Table S2: summary of sample sizes, quality scores, hazard ratios, confidence intervals, *p*-values, and treatment responses of the primary and secondary endpoints reported in the studies.
