# Supplementary material for: Prognostic Biomarkers for Survival in Nasopharyngeal Carcinoma: A Systematic Review of the Literature
Source: Cancers (Basel). 2022 Apr 24;14(9):2122. doi: 10.3390/cancers14092122 (PMC9103785; doi:10.3390/cancers14092122)
Supplement: Supplementary file 1 [file cancers-14-02122-s001.zip › cancers-1694596-supplementary.pdf]

<u>Table S1:</u> Literature search parameters on PubMed. The advanced search tool in PubMed was utilized to scan for all the studies relevant to the search parameters from 2011 to 2021. Both Chinese and English languages were accepted. The search resulted in 460 items being returned for further screening and analysis.

Criteria	Search term*
Diseases	(("nasopharyn*"[Title] AND ("carcinoma"[Title] OR "cancer"[Title] OR "neoplasm*"[Title])) OR ("NPC"[Title] AND "nasopharyn*"[Text Word]))
Theme	("stag*"[Title/Abstract] OR "TNM"[Title/Abstract] OR "prognos*"[Title/Abstract]) AND ("biomark*"[Title/Abstract])
Language	("Chinese"[Language] OR "English"[Language])
Publication date	(2011:2021[pdat])
Items returned	460

*The literature search was performed on 9 September 2021.

<u>Table S2</u>: Summary of sample sizes, quality scores, hazard ratios, confidence intervals, P-values and treatment responses of the primary and secondary endpoints reported in the studies. The primary and secondary endpoints, along with their relevant data elements were extracted from the selected studies and systematically recorded. The HRs and P-values of each of the survival types reported in the studies depict that they are significant and independent of the multivariate clinical parameters. The high quality scores signify the low risk of bias present in the studies.

Author, Year of publication	Prognostic factor	Validation sample size	Independent sample size	Multivariate clinical parameters	Survival type	HR[1]	95% CI	P-value	Treatment response (HR, 95% CI, P-value)	Quality score
Guo et al., 2015 [1]	rs3740194 in *CELF2* gene	1520	2237[2]	1. Gender (male vs female)<br>2. Age (>=50 vs. <50 years)<br>3. T status (T3-T4 vs T1-T2)<br>4. N status (N2-N3 vs N0-N1)<br>5. Radiotherapy (3D-RT vs 2D-RT)<br>6. ICT (yes vs no)<br>7. CCT (yes vs no)	OS	1.53	1.23 – 1.89	1.30×10^{-4}	2D-RT only (1.88, 1.27-2.81, 0.002), 2D-RT plus CCT (1.60, 1.13-2.26, 0.007)	15

[1] Adjusted
[2] Combined set

				8. ACT (yes vs no)<br>9. *CELF2* rs3740194 (AA vs. AG)<br>10. *CELF2* rs3740194 (AA vs. GG)<br>11. *CELF2* rs3740194 (AA vs AG+GG)	DMFS	1.60	1.26 – 2.02	8.87 x 10^{-5}		
Guo et al., 2020 [2]	rs1131636 in *RPA1* gene	1751	545[3]	1. Gender (female vs. male)<br>2. Age (>=50 vs. <50)<br>3. Clinical stage (advanced vs. early)<br>4. IMRT (yes vs. no)<br>5. ICT (yes vs. no)<br>6. CCRT (yes vs. no)<br>7. ACT (yes vs. no)<br>8. rs1131636 (additive model)	OS<br>DMFS	1.33<br>1.16	1.20 – 1.47<br>1.05 – 1.28	6.31 x 10^{-8}<br>0.0033	No data available	15
Liu et al., 2012 [3]	miR-142-3p, miR-29c, miR-26a, miR-30e, miR-93	156	153	1. miRNA signature (high risk vs. low risk)<br>2. Sex (male vs. female)<br>3. Age (<45 vs >=45)<br>4. TNM stage (stage III-IV vs I-II)	OS	3.07	1.34 – 7.01	0.0082	CCT, yes vs. no; low-risk group (0.25, 0.08-0.74, 0.013)	15

[3] Validation cohort from Singapore

				5. WHO pathological type (undifferentiated non-keratinizing vs differentiated non-keratinizing vs keratinizing squamous cell)<br>6. Radiotherapy period interruptions (0 days vs. >=1 day)<br>7. Radiotherapy boosting (no vs. yes)<br>8. CCT (yes vs no)<br>9. VCA IgA (<1:80 vs. 1:80-1:320 vs 1:640)<br>10. EA IgA (<1:10 vs. 1:10-1:20 vs >=1.40)	DMFS	2.39	1.05 – 5.42	0.037	CCT, yes vs. no; low-risk group (0.30, 0.10-0.91, 0.033)	
					DFS	3.16	1.65 – 6.04	0.0011	CCT, yes vs. no; low-risk group (0.25, 0.09-0.68, 0.007)	
Liu et al., 2014 [4]	miR-22, miR-572, miR-638, miR-1234	256	512[4]	1. miRNA signature (high risk vs. low risk)<br>2. TNM stage (III–IV vs. I–II)	OS	2.40	1.71 – 3.37	<0.001	No data available	15
					DMFS	3.31	2.18 – 5.02	<0.001		
Ren et al., 2017 [5]	*HOPX* hypermethylation	188		1. Methylation level (high vs. low)<br>2. Age (>=45 vs. <45)<br>3. Sex (male vs. female)<br>4. TNM stage (III-IV vs. 1-II)<br>5. WHO type (III vs. I+II)<br>6. VCA IgA (>=80 vs <80)<br>7. EA IgA (>=10 vs <10)	OS	2.13	1.15 – 3.93	0.016	No data available	15
					DMFS	2.75	1.26 – 6.03	0.011		

[4] Combined set

					DFS	1.96	1.12 – 3.43	0.019		
Ren et al., 2018 [6]	TIPE3 hypermethylation	187		1. Methylation level (high vs. low)<br>2. Age (>45 vs. <=45)<br>3. Sex<br>4. TNM stage (III-IV vs. I-II)<br>5. WHO type<br>6. VCA IgA<br>7. EA IgA	OS	1.80	1.02 – 3.16	0.041	No data available	15
					DMFS	2.69	1.21 – 5.96	0.015		
					DFS	1.71	1.01 – 2.89	0.045		
Jiang et al., 2015 [7]	Hypermethylated gene panel (*WIF1*, *UCHL1*, *RASSF1A*, *CCNA1, TP73, SFRP1*)	150	153	1. Methylated gene panel (high vs low)<br>2. Sex<br>3. Age (<45 vs >=45)<br>4. TNM stage (IV vs. III vs. II vs. I)<br>5. WHO pathology type (undifferentiated nonkeratinizing vs. differentiated keratinizing)<br>6. CCT (yes vs. no)	OS	1.83	1.01 – 3.31	0.046	CCT, yes vs. no; low methylation (0.45, 0.22-0.93, 0.031)	15
					DFS	2.08	1.17 – 3.68	0.013	CCT, yes vs. no; low methylation (0.58, 0.28-0.96, 0.045)	
Dai et al., 2020 [8]	COSMIC mutational signatures	113		1. Stage (IV vs I-II)<br>2. Age	OS	1.9	1.0 – 3.7	0.046	No relevant	15

	(HR & MMR		402[5]	3. Gender (male vs. female)<br>4. *BRCA2* germline variants (Positive vs. negative)	PFS[6]	1.9	1.0 – 3.4	0.042	data available	
Wang et al., 2011 [9]	Eight-signature classifier (patient sex, *EBV-LMP1*, CD14, caveolin-1, p-P70S6K, *MMP11*, survivin, *SPARC*)	1059		1. NPC-SVM Classifier (High risk vs. low risk)<br>2. Age (>46 vs <=46)<br>3. Clinical stage (III-IV vs I-II)<br>4. WHO classification (keratinizing squamous cell carcinoma and non-keratinizing differentiated carcinoma vs. non-keratinizing undifferentiated carcinoma)	DSS	4.9	3.0 – 7.9	<0.001	No data available	15
Liu et al., 2020 [10]	Immune signature (PD-L1+CD163+, *CXCR5*, CD117)	304		1. Immune signature (high vs. low risk)<br>2. Gender (male vs. female)<br>3. Age (>=45 vs <45)<br>4. T stage (T3-4 vs T1-2)<br>5. N stage (N2-3 vs N0-1)<br>6. Overall stage (I-III vs IV),<br>7. ECOG score (0 vs 1 vs 2)	DMFS	4.297	2.182 – 8.461	<0.001	IC+CCRT vs. CCRT; low-risk (0.355, 0.147 - 0.857, 0.021)	15

[5] Prospective

[6] Independent cohort analysis

				8. LDH (>=245 vs <245 U/L)<br>9. CRP (>=3 vs <3 mg/L)<br>10. Hg (>=130 vs <130 g/L)<br>11. BMI (>=23 vs <23 kg/m2)	PFS	2.115	1.289 – 3.469	0.003	IC+CCRT vs. CCRT; low-risk (0.590, 0.351 - 0.992, 0.047)	
Zhang et al., 2013 [11]	*DLL4* & VEGF expression	113	444[7]	1. *DLL4* expression (high vs low)<br>2. Age (> 47 vs <=47)<br>3. Gender (male vs female)<br>4. TNM stage (III+IV vs I+II)	DSS[8]	1.809	1.380 – 2.370	<0.001	No data available	15
Wang et al., 2018 [12]	Tumour-infiltrating lymphocytes (TILs)	584	304	1. TILs (low vs high)<br>2. T stage (T2, T3, T4 vs. T1)<br>3. N stage (N2, N3 vs. N0-N1)<br>4. Radiotherapy technique (IMRT vs 2DRT/3DRT)<br>5. EBV DNA (>=4000 copy/mL vs. <4000 copy/mL)<br>6. LDH (>=245 U/L vs. <245 U/L)	OS[9]	0.52	0.32 – 0.84	0.008	No data available	15
					DMFS[9]	0.47	0.27 – 0.82	0.007		
					DFS	0.52	0.34 – 0.79	0.002	IMRT vs. 2DRT/3DRT (0.37, 0.23-0.60, <0.000)	

[7] Combined set
[8] Overall cases
[9] Univariate analysis

Tang et al., 2018 [13]	13 gene-signature (*YBX3, CBR3, CXCL10, CLASP1, DCTN1, FNDC3B, WSB2, LRIG1, GRM4, ANXA1, WNK1, HDLBP, POLR2M*)	204	165	1. DMGN (high vs. low)<br>2. Gender (male vs. female)<br>3. Age (>=45 vs <45)<br>4. N-stage (N2-3 vs N0-1)<br>5. T-stage (T3-4 vs T1-2)<br>6. PET-CT (no vs. yes)<br>7. VCA-IgA (>= 1:80, vs <1:80)<br>8. EA-IgA (>=1:10 vs. <1:10)<br>9. LDH (>=245 vs. <245 U/L)<br>10. CRP (>=8.2 vs. <8.2 mg/L)<br>11. Hg (>=120 vs. <120 g/L)<br>12. BMI (>=18.5 vs <18.5 kg/m3)<br>13. Chemotherapy (no vs. yes)	OS	2.05	1.23 – 3.43	0.0061	CCT, yes vs. no; low risk (0.58, 0.35-0.97, 0.036)	15
					DMFS	3.32	1.74 – 6.33	0.00028	CCT, yes vs. no; low risk (0.40, 0.19-0.83, 0.011)	
					DFS	2.35	1.40 – 3.95	0.0012	CCT, yes vs. no; low risk (0·56, 0·34-0·94, 0·025)	

Gao et al., 2017 [14]	Protein tyrosine phosphatase 4A2 (*PTP4A2*)	201	266[10]	1. Age (>=45 vs <45)<br>2. Gender (male vs female)<br>3. T status (T3-4 vs T1-2)<br>4. N status (N2-3 vs N0-1)<br>5. TNM stage (III-IV vs I-II)<br>6. WHO type (Type III vs Type I-II)<br>7. VCA IgA (>=1:80 vs <1:80)<br>8. EA IgA (>=1:10 vs <1:10)	OS	5.065	3.490 – 7.351	<0.001	No data available	15
					DFS	3.669	2.635 – 5.110	<0.001		

HR, hazard ratio; CI confidence interval; OS, overall survival; DMFS, distant-metastasis free survival; DFS, diseases-free survival; PFS, progression-free survival; DSS, disease-specific survival; RT, radiotherapy; IMRT, intensity modulated radiotherapy; ICT, induction chemotherapy; CCT, concurrent chemotherapy; CCRT, concurrent chemoradiotherapy; ICT, induction chemotherapy; VCA IgA, viral capsid antigen immunoglobin-A; EA IgA, early antigen immunoglobin-A; ECOG, Eastern Cooperative Oncology Group; LDH, lactate dehydrogenase; CRP, C-reactive protein; Hg, hemoglobin; BMI, body mass index; PET-CT, Positron Emission Tomography and Computed Tomography; DMGN, distant metastasis gene signature

References

1. Guo, Y. M., M. X. Sun, J. Li, T. T. Liu, H. Z. Huang, J. R. Chen, W. S. Liu, Q. S. Feng, L. Z. Chen, J. X. Bei, and Y. X. Zeng. "Association of Celf2 Polymorphism and the Prognosis of Nasopharyngeal Carcinoma in Southern Chinese Population." *Oncotarget* 6, no. 29 (2015): 27176-86.
2. Guo, Y. M., J. R. Chen, Y. C. Feng, M. L. K. Chua, Y. Zeng, E. P. Hui, A. K. C. Chan, L. Q. Tang, L. Wang, Q. Cui, H. Q. Han, C. L. Luo, G. W. Lin, Y. Liang, Y. Liu, Z. L. He, Y. X. Liu, P. P. Wei, C. J. Liu, W. Peng, B. W. Han, X. Y. Zuo, E. H. W. Ong, E. L. L. Yeo, K. P. Low, G. S. Tan, T. K. H. Lim, J. S. G. Hwang, B. Li, Q. S. Feng, X. Xia, Y. F. Xia, J. Ko, W. Dai, M. L. Lung, A. T. C. Chan, D. Y. M. Lo, M. S. Zeng, H. Q. Mai, J. Liu, Y. X. Zeng, and J. X. Bei. "Germline Polymorphisms and Length of Survival of Nasopharyngeal Carcinoma: An Exome-Wide Association Study in Multiple Cohorts." *Adv Sci (Weinh)* 7, no. 10 (2020): 1903727.

[10] Overall cases

3. Liu, N., N. Y. Chen, R. X. Cui, W. F. Li, Y. Li, R. R. Wei, M. Y. Zhang, Y. Sun, B. J. Huang, M. Chen, Q. M. He, N. Jiang, L. Chen, W. C. Cho, J. P. Yun, J. Zeng, L. Z. Liu, L. Li, Y. Guo, H. Y. Wang, and J. Ma. "Prognostic Value of a Microrna Signature in Nasopharyngeal Carcinoma: A Microrna Expression Analysis." *Lancet Oncol* 13, no. 6 (2012): 633-41.
4. Liu, N., R. X. Cui, Y. Sun, R. Guo, Y. P. Mao, L. L. Tang, W. Jiang, X. Liu, Y. K. Cheng, Q. M. He, W. C. Cho, L. Z. Liu, L. Li, and J. Ma. "A Four-Mirna Signature Identified from Genome-Wide Serum Mirna Profiling Predicts Survival in Patients with Nasopharyngeal Carcinoma." *Int J Cancer* 134, no. 6 (2014): 1359-68.
5. Ren, X., X. Yang, B. Cheng, X. Chen, T. Zhang, Q. He, B. Li, Y. Li, X. Tang, X. Wen, Q. Zhong, T. Kang, M. Zeng, N. Liu, and J. Ma. "Hopx Hypermethylation Promotes Metastasis Via Activating Snail Transcription in Nasopharyngeal Carcinoma." *Nat Commun* 8 (2017): 14053.
6. Ren, X. Y., X. Wen, Y. Q. Li, J. Zhang, Q. M. He, X. J. Yang, X. R. Tang, Y. Q. Wang, P. P. Zhang, X. Z. Chen, B. Cheng, J. Ma, and N. Liu. "Tipe3 Hypermethylation Correlates with Worse Prognosis and Promotes Tumor Progression in Nasopharyngeal Carcinoma." *J Exp Clin Cancer Res* 37, no. 1 (2018): 227.
7. Jiang, W., N. Liu, X. Z. Chen, Y. Sun, B. Li, X. Y. Ren, W. F. Qin, N. Jiang, Y. F. Xu, Y. Q. Li, J. Ren, W. C. Cho, J. P. Yun, J. Zeng, L. Z. Liu, L. Li, Y. Guo, H. Q. Mai, M. S. Zeng, T. B. Kang, W. H. Jia, J. Y. Shao, and J. Ma. "Genome-Wide Identification of a Methylation Gene Panel as a Prognostic Biomarker in Nasopharyngeal Carcinoma." *Mol Cancer Ther* 14, no. 12 (2015): 2864-73.
8. Dai, W., D. L. Chung, L. K. Chow, V. Z. Yu, L. C. Lei, M. M. Leong, C. K. Chan, J. M. Ko, and M. L. Lung. "Clinical Outcome-Related Mutational Signatures Identified by Integrative Genomic Analysis in Nasopharyngeal Carcinoma." *Clin Cancer Res* 26, no. 24 (2020): 6494-504.
9. Wang, H. Y., B. Y. Sun, Z. H. Zhu, E. T. Chang, K. F. To, J. S. Hwang, H. Jiang, M. K. Kam, G. Chen, S. L. Cheah, M. Lee, Z. W. Liu, J. Chen, J. X. Zhang, H. Z. Zhang, J. H. He, F. L. Chen, X. D. Zhu, M. Y. Huang, D. Z. Liao, J. Fu, Q. Shao, M. B. Cai, Z. M. Du, L. X. Yan, C. F. Hu, H. K. Ng, J. T. Wee, C. N. Qian, Q. Liu, I. Ernberg, W. Ye, H. O. Adami, A. T. Chan, Y. X. Zeng, and J. Y. Shao. "Eight-Signature Classifier for Prediction of Nasopharyngeal [Corrected] Carcinoma Survival." *J Clin Oncol* 29, no. 34 (2011): 4516-25.
10. Liu, S. L., L. J. Bian, Z. X. Liu, Q. Y. Chen, X. S. Sun, R. Sun, D. H. Luo, X. Y. Li, B. B. Xiao, J. J. Yan, Z. J. Lu, S. M. Yan, L. Yuan, L. Q. Tang, J. M. Li, and H. Q. Mai. "Development and Validation of the Immune Signature to Predict Distant Metastasis in Patients with Nasopharyngeal Carcinoma." *J Immunother Cancer* 8, no. 1 (2020).
11. Zhang, J. X., M. B. Cai, X. P. Wang, L. P. Duan, Q. Shao, Z. T. Tong, D. Z. Liao, Y. Y. Li, M. Y. Huang, Y. X. Zeng, and J. Y. Shao. "Elevated Dll4 Expression Is Correlated with Vegf and Predicts Poor Prognosis of Nasopharyngeal Carcinoma." *Med Oncol* 30, no. 1 (2013): 390.
12. Wang, Y. Q., Y. P. Chen, Y. Zhang, W. Jiang, N. Liu, J. P. Yun, Y. Sun, Q. M. He, X. R. Tang, X. Wen, X. J. Yang, P. P. Zhang, J. Zhang, Y. Lei, Y. Q. Li, and J. Ma. "Prognostic Significance of Tumor-Infiltrating Lymphocytes in Nondisseminated Nasopharyngeal Carcinoma: A Large-Scale Cohort Study." *Int J Cancer* 142, no. 12 (2018): 2558-66.
13. Tang, X. R., Y. Q. Li, S. B. Liang, W. Jiang, F. Liu, W. X. Ge, L. L. Tang, Y. P. Mao, Q. M. He, X. J. Yang, Y. Zhang, X. Wen, J. Zhang, Y. Q. Wang, P. P. Zhang, Y. Sun, J. P. Yun, J. Zeng, L. Li, L. Z. Liu, N. Liu, and J. Ma. "Development and Validation of a Gene Expression-Based Signature to Predict Distant Metastasis in Locoregionally Advanced Nasopharyngeal Carcinoma: A Retrospective, Multicentre, Cohort Study." *Lancet Oncol* 19, no. 3 (2018): 382-93.

14. Gao, Y., M. Zhang, Z. Zheng, Y. He, Y. Zhu, Q. Cheng, J. Rong, H. Weng, C. Chen, Y. Xu, M. Yun, J. Zhang, and S. Ye. "Over-Expression of Protein Tyrosine Phosphatase 4a2 Correlates with Tumor Progression and Poor Prognosis in Nasopharyngeal Carcinoma." *Oncotarget* 8, no. 44 (2017): 77527-39.
